# Non-Enzymatic Modification of Aminophospholipids by Carbonyl-Amine Reactions

**DOI:** 10.3390/ijms14023285

**Published:** 2013-02-05

**Authors:** Alba Naudí, Mariona Jové, Victòria Ayala, Rosanna Cabré, Manuel Portero-Otín, Reinald Pamplona

**Affiliations:** Department of Experimental Medicine, Faculty of Medicine, University of Lleida-Biomedical Research Institute of Lleida (UdL-IRBLleida), Lleida E25198, Catalonia, Spain; E-Mails: alba.naudi@mex.udl.cat (A.N.); mariona.jove@udl.cat (M.J.); victoria.ayala@mex.udl.cat (V.A.); rosanna.cabre@mex.udl.cat (R.C.); manuel.portero@mex.udl.cat (M.P.-O.)

**Keywords:** advanced glycation endproducts, advanced lipoxidation endproducts, age-associated diseases, aging, cell membrane, carbonyl compounds, lipid peroxidation, Maillard reaction products, oxidative stress, phosphatidylethanolamine, phosphatidylserine

## Abstract

Non-enzymatic modification of aminophospholipids by lipid peroxidation-derived aldehydes and reducing sugars through carbonyl-amine reactions are thought to contribute to the age-related deterioration of cellular membranes and to the pathogenesis of diabetic complications. Much evidence demonstrates the modification of aminophospholipids by glycation, glycoxidation and lipoxidation reactions. Therefore, a number of early and advanced Maillard reaction-lipid products have been detected and quantified in different biological membranes. These modifications may be accumulated during aging and diabetes, introducing changes in cell membrane physico-chemical and biological properties.

## 1. Introduction

Life demands membranes. Biological membranes are dynamic structures that generally consist of amphipathic molecules bilayers held together by non-covalent bonds [1,2]. In eukaryotic cells, phospholipids are the predominant membrane lipids and are, from a topographic point of view, asymmetrically distributed across the bilayer [3–5]. Phospholipids consist of a hydrophilic head group with attached hydrophobic acyl chains. The variation in head groups and aliphatic chains allows the existence of more than 1000 different phospholipid species in any eukaryotic cell [6,7]. Phosphatidylcholine (PC), phosphatidylethanolamine (PE), phosphatidylserine (PS), phosphatidylinositol (PI) and cardiolipin (CL), as well as sphingomyelin (SM) and glycosphingolipids (GS) are the major phospholipid classes [1,2]. In most eukaryotic membranes, PC and PE represent together around 60%–85% of the phospholipid fraction, while the fraction of other phospholipids depends on the cell membrane and even on animal species [1,2,8,9]. Phospholipids play multiple roles. They constitute a permeability barrier, modulate the functional properties of membrane-associated activities, provide a matrix for the assembly and function of a wide variety of catalytic processes, and act as donors during the synthesis of macromolecules. The wide range of processes in which phospholipids are specifically involved explains the need for diversity in phospholipid structures and fatty acid composition [6,10]. This diversity requires complex metabolic and regulatory pathways [1,2]. Therefore, for example, eukaryotic cells invest around 5% of their genes to synthesize all of these lipids [9].

The various phospholipid classes that comprise cell membranes are distributed over both leaflets of the bilayer in a non-random fashion [3–5]. This is especially evident for the aminophospholipids, PS and PE, which preferentially reside in the plasma membranes’ inner leaflet [11,12]. Where does lipid asymmetry originate, and how is it maintained and regulated? What is the functional role of aminophospholipid asymmetry, and what are the consequences of the breakdown of regulatory processes that result in the exposure of aminophospholipids in the cells’ outer leaflet? Although these key questions mainly remain unanswered, recent studies have established that cells have developed a number of mechanisms to deal with this issue. Targeting phospholipids to specific membrane sites is essential for maintaining critical signal transduction cascades, cell shape, hemostasis, and homeostasis [12]. In particular, aminophospholipids have been implicated in a diverse array of processes ranging from cell proliferation to cell death, from catabolism to inflammation [12]. In this scenario, asymmetry is maintained by active ATP-dependent processes, suggesting that is critical to normal cell function. Specifically, aminophospholipid asymmetry is controlled by one or more specific mechanisms, which involve selective interactions between lipids and cytoskeletal proteins and an aminophospholipid-specific active transport system [11,12].

The acyl chains are composed of either saturated, monounsaturated or polyunsaturated hydrocarbon chains that normally vary from 14 to 22 carbons in length [13]. In eukaryotic cells from vertebrate species, the average chain length of a biological membrane is strictly maintained by around 18 carbon atoms, and the relative distribution between saturated and unsaturated fatty acids follows the ratio 40:60 [14]. Polyunsaturated fatty acids (PUFAs) are essential components of cellular membranes in higher eukaryotes that strongly affect their fluidity, flexibility and selective permeability. Additionally, PUFAs affect many cellular and physiological processes in animals, including modulation of ion channels and carriers, activities of membrane-associated enzymes, and regulation of gene expression, among others [13].

Thus, membrane composition (phospholipids classes’ distribution and fatty acid profile) is strictly and dynamically regulated. The mechanisms of the homeostatic regulation of the membrane composition, the mechanisms that create lipid asymmetry and their functional implications, and the full definition of the utility of the eukaryotic lipid repertoire are beginning to be understood, being an exciting and rapidly expanding field.

## 2. Membrane Unsaturation and Lipid Peroxidation

As a principle, chemical reactions in living cells are under strict enzyme control and are tightly regulated by the metabolic program. One of the attractors involved in biomolecular evolution is the minimizing of unnecessary side reactions. Nevertheless, uncontrolled and potentially deleterious reactions occur, even under physiological conditions. Oxidative damage is a broad term used to cover the attack upon biological molecules by free radicals—chemical species with one unpaired electron. Free radicals attack/damage all cellular constituents [14]. In this context, the susceptibility of membrane phospholipids to oxidative damage is related to two inherent traits, the physico-chemical properties of the membrane bilayer and the chemical reactivity of the fatty acids composing the membrane [15]. The first property is related to the fact that oxygen and free radicals are more soluble in the fluid lipid bilayer than in the aqueous solution. Thus, membranes contain an interior organic phase, in which oxygen may tend to concentrate. Therefore, these differences in solubility are important when considering the availability of oxygen/free radicals for chemical reactions inside living systems: Organic regions may contain more free radicals than aqueous regions [16,17] and, consequently, membrane lipids become primary targets of oxidative damage. The second property is related to the fact that PUFA residues of phospholipids are extremely sensitive to oxidation. Every membrane phospholipid contains an unsaturated fatty acid residue esterified to the 2-hydroxyl group of its glycerol moiety. Many of these are polyunsaturated and the presence of a methylene group between two double bonds renders the fatty acid sensitive to free radical-induced damage, their sensitivity to oxidation increasing exponentially as a function of the number of double bonds per fatty acid molecule [18,19]. Consequently, the high concentration of PUFAs in phospholipids not only makes them prime targets for reaction with oxidizing agents but also enables them to participate in long free radical chain reactions.

**Figure 1:**
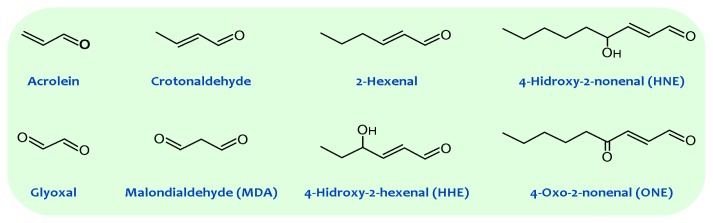
General structures of principal reactive carbonyl species detected in biological systems.

Lipid peroxidation generates hydroperoxides as well as endoperoxides, which undergo fragmentation to produce a broad range of reactive intermediates called reactive carbonyl species (RCS) with three to nine carbons in length (see Figure 1), the most reactive being α,β-unsaturated aldehydes (4-hydroxy-trans-2-nonenal (HNE) and acrolein), di-aldehydes (malondialdehyde (MDA) and glyoxal), and keto-aldehydes (4-oxo-trans-2-nonenal (ONE) and isoketals) [20,21]. 2-Hydroxyheptanal (2-HH) is another major aldehydic product of lipid peroxidation of PUFAn-6, while 4-hydroxyhexenal (4-HHE) is generated in a lower yield. Additionally, a number of other short chain aldehydes are produced during lipid peroxidation through poorly understood mechanisms. These carbonyl compounds, ubiquitously generated in biological systems, have unique properties contrasted with free radicals [14]. For instance, compared with free radicals, reactive aldehydes have a much longer half-life (*i.e.*, minutes to hours instead of microseconds to nanoseconds for most free radicals). Further, the non-charged structure of aldehydes allows them to migrate with relative ease through hydrophobic membranes and hydrophilic cytosolic media, thereby extending the migration distance far from the production site. Based on these features alone, these carbonyl compounds can be more destructive than free radicals and may have far-reaching damaging effects on target sites within or outside membranes.

Therefore, the highly unsaturated fatty acids present in cellular membranes are the most susceptible macromolecules to oxidative damage in cells, and this sensitivity increases as a function of their number of double bonds. In addition, the carbonyl compounds generated as lipid peroxidation-derived end products extend the membrane damage to other cellular constituents.

## 3. Non-Enzymatic Modification of Cellular Components: The Maillard Reaction-Derived Molecular Damage

Carbonyl compounds react with nucleophilic groups in macromolecules like proteins and DNA, resulting in their chemical, non-enzymatic, and irreversible modification and finally in the formation of a variety of adducts and cross-links collectively named Advanced Lipoxidation Endproducts (ALEs) [22,23]. Thus, by reacting with nucleophilic sites in proteins (belonging basically to Cys, Lys, Arg, and His residues), carbonyl compounds generate ALE adducts such as MDA-Lys, HNE-Lys, FDP-Lys, and *S*-carboxymethyl-cysteine; and the cross-links glyoxal-lysine dimmer, and lysine-MDA-lysine, among several others. The accumulation of MDA adducts on proteins is also involved in the formation of lipofuscin. Thus, lipofuscin becomes a nondegradable intralysosomal fluorescent pigment formed through lipoxidative reactions [24]. In addition to proteins, lipid peroxidation-derived endproducts can also react with the exocyclic amino groups of deoxyguanosine, deoxyadenosine, and deoxycytosine to form various alkylated products [25]. Guanine is, however, the most commonly modified DNA base because of its high nucleophilicity. Some common enals that cause DNA damage, analogously to proteins, are MDA, HNE, and acrolein, among others. Thus, the most common adducts arising from enals are exocyclic adducts such as etheno adducts, and MDA-deoxyguanosine (M1dG).

In addition to lipid peroxidation derived carbonyl compounds, reducing sugars and carbonyl compounds derived from carbohydrate oxidation can also react with the primary amino groups of macromolecules such as proteins and DNA, following the principles of the carbonyl-amine reaction (also named Maillard reaction) [22]. The early Schiff base and Amadori adducts (glycation reaction), which form subsequently, slowly undergo a succession of intramolecular rearrangements, dehydration, and oxidation-reduction reactions to produce the terminal products termed advanced glycation end products (AGEs), which are often chemically irreversible, thus persisting throughout the life of the affected macromolecule [26,27]. More important, a major spin-off of studies on glycation during the 1980s was the recognition that oxidative reactions, and by inference, oxidative stress, catalyzed the chemical modification of proteins and DNA by Maillard reactions *in vivo* [28].

**Figure 2:**
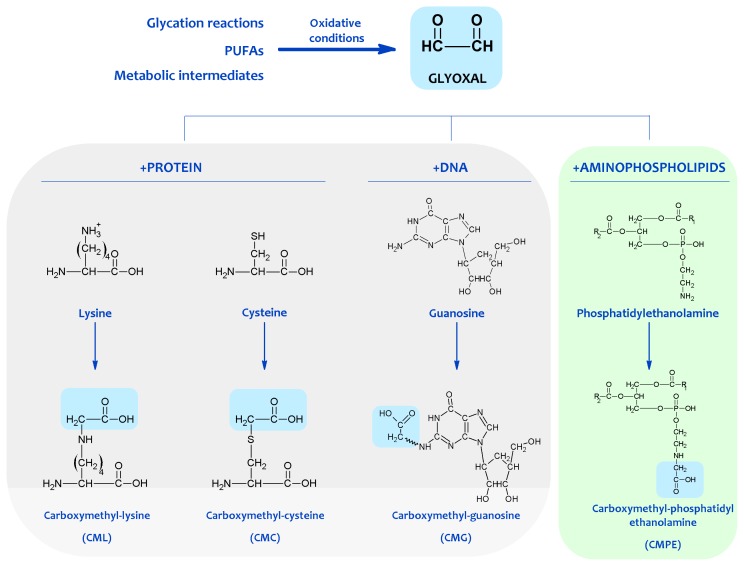
Protein, DNA and aminophospholipid damage resulting from carbonyl products of lipid peroxidation. Shown are examples of molecular adducts (Advanced Lipoxidation Endproducts, ALEs) generated by the reactive carbonyl compound glyoxal.

In this scenario, it is likely that the amino group of aminophospholipids will also react with carbonyl compounds and initiates some of the reactions occurring in proteins and DNA, expanding the biological effects of the carbonyl-amine or Maillard reaction (see Figure 2).

Hence, Maillard reaction-derived molecular damage is a natural consequence of aerobic life. Maillard reaction products (MRPs, including AGEs and ALEs) induce the chemical, non-enzymatic and irreversible modification of cellular constituents, and they are part of the evidence for the existence of an “oxidative stress” *in vivo*. Little is known about molecular modification by MRPs, particularly regarding aminophospholipids. A current challenge is to establish the chemical structure of these modifications and the mechanisms for their formation, and to identify which factors control the nature, selectivity, extent, irreversibility and biological-pathological consequences of the molecular modification occurring *in vivo*.

## 4. Chemical Modification of Aminophospholipids by Carbonyl-Amine Reactions

Recent reports indicate that, like proteins and DNA, aminophospholipids are also targets of Maillard-type reactions. In early studies, the formation of products resulting from the reaction between aminophospholipids and lipoperoxidation-derived aldehydes such as MDA and HNE were described, but the exact structure of the products was not established. In these works, it was established that the amount of free amino groups significantly decreased during oxidation of phospholipids, developing a brownish-yellow color attributed to a Maillard-type reaction [29]. It was also reported that free amino groups of PE disappeared during oxidation in proportion to the oxygen absorbed [30]. Probably, both findings can be attributed to the reaction of the PE amino group with carbonyls, mainly MDA produced by lipid oxidation, leading to Schiff base formation as assessed by fluorescence. So, during peroxidation, PE and PS formed fluorescent chromophores with maximum emission ranging from 440 to 490 nm and maximum excitation between 360 and 400 nm [31,32]. Fluorescence development was related to (i) formation of thiobarbituric acid reactive substances (TBARS), especially MDA; (ii) reaction time; (iii) availability of reactive amino groups on the aminophospholipids, and (iv) antioxidant (alpha-tocopherol) content in an inverse fashion. These chromophores showed similarities to those formed in model membranes [33] or in rat liver mitochondrial and microsomal fractions peroxidized *in vitro* [31,32]. Furthermore, lipid extracts isolated from lipofuscin [34] and from tissues of lipid peroxidation-experimental models such as old, vitamin E deficient animals or animals stressed with highly unsaturated lipid diets, showed similar fluorescence properties [31,32]. Table 1 offers, chronologically, a summary of studies dedicated to the characterization of Maillard reaction-derived compounds on aminophospholipids.

Using thin-layer chromatography (TLC), non-enzymatic modification of aminophospholipids by lipoperoxidation derived products were also detected in red blood cells (RBC) [35–42] and in eye lens membranes [43,44]. These modifications corresponded to a Schiff’s base adduct formed by cross-linking the PE and PS amino with MDA aldehyde groups, based on the following evidence: (i) A new lipid spot appeared between PS and PE; (ii) its intensity was proportional to MDA concentration both *in vivo* and *in vitro*; (iii) in selective staining procedures, it was phosphorus positive and ninhydrin negative; (iv) when this compound was exposed to acid vapors and then developed in a second direction, the “adduct” was resolved into two equimolar spots of PS and PE which were ninhydrin positive; (v) other non-amino phospholipids were ineffective in the formation of this compound; (vi) its fluorescence characteristics were compatible with a Schiff base conjugate formed between MDA and aminophospholipids; and finally, (vii) added antioxidants, blocking MDA formation, avoided its appearance.

Lipid peroxidation leads to the formation of other aldehydes, such as HNE, able to react with aminophospholipids. Accordingly, the formation of fluorescent chromolipids was detected when HNE was incubated with aminophospholipids, microsomes and mitochondrial fractions [45,46]. Spectral characteristics of these chromolipids showed excitation maxima at 350–360 nm and emission at 430 nm, with the fluorescence intensity being linearly related to the number of HNE molecules reacting with aminophospholipids [45]. More recently, HNE potential capacity to react with aminophospholipids has been reported. Thus, by using TLC-high performance liquid chromatography (HPLC)-liquid chromatography (LC)-mass spectrometry (MS) techniques, it has been found that the main resulting compounds were a Michael adduct plus a minor Schiff base adduct, partly cyclized as a pyrrole derivative via a loss of water, with PE being more reactive than PS [46].

**Figure 3:**
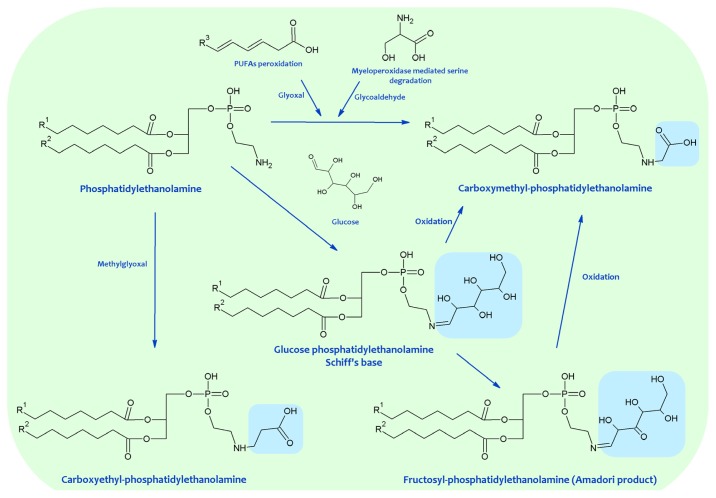
Advanced glycation end products (AGEs)-lipid products resulting from the Maillard reaction.

Much evidence demonstrates the *in vitro* and *in vivo* occurrence of the Schiff base, Amadori and AGEs-lipid products resulting from the Maillard reaction (see also Table 1 and Figure 3). The Schiff base formation between glucose and aminophospholipids was documented in experimental models and in human RBC membranes, plasma, and low-density lipoproteins (LDL) [53,77–79]. The existence of glycated aminophospholipids in its Schiff base form was confirmed by using HPLC LC-electrospray ionization (ESI)-MS. Reduction with NaBH_3_CN, shifting the retention time and increasing the detected mass of glycated lipids by two units, confirmed the identity of the major analyte as a Schiff base. Surprisingly, only the diacyl species became glycated and neither the alkylacyl nor the alkenylacyl were modified; furthermore, in contrast with *in vitro* experiments, PS glycation was not detected.

*In vitro* model reactions of d-glucose and PE demonstrated the formation of Amadori products, which were also detected *in vivo* [55–58,73]. Chromatographic and spectroscopic characterization unequivocally proved the existence of deoxy-d-fructosyl PE [55,56]. The formation of the Amadori product has also been demonstrated through the synthesis and characterization of *N*-(glucitol)ethanolamine (GE), a stable reduction product of glycated PE, detected in RBC membranes [58]; and by 5-(hydroxymethyl)-2-furfuraldehyde formation from the acid-treated phospholipid fraction as a stable derivative of an Amadori product, in this case being detected in rat liver lipid extracts [73].

Early immunological studies indicated the presence of MRPs covalently attached to aminophospholipids such as PE in LDL, although their structures remain unknown [72]. In addition, by using an ELISA assay, it was concluded that the majority of MRPs present in LDL were localized in the lipid phase. Nevertheless, subsequent analyses identified carboxymethyl-lysine (CML) as the major AGE antigen in tissue proteins, and it was proposed that AGE-lipids could be actually immunologically cross-reactive carboxymethyl (CM) derivatives of PE and PS formed by phospholipid glyco- and lipoxidation, similarly to proteins [80]. So, by using a selected selective ion monitoring-gas chromatography-Mass spectrometry (SIM-GC-MS) assay for carboxymethylethanolamine (CME) [54,74,75,81] and carboxymethylserine (CMS) [58], the hydrolysis products of CM-PE and CM-PS, respectively, it was possible to demonstrate CME and CMS formation *in vitro* during the glycation of dioleoyl-PE under air and from linoleoylpalmitoyl-PE, but not from dioleoyl-PE, in absence of glucose. These data seem to indicate that carboxymethylation may proceed either from glucose or PUFA under oxidizing conditions. In experimental models, glucose, which is more resistant to autoxidation than PUFAs, was present at a 33-fold molar excess over free amino groups, whereas PUFA (linoleic acid) in PE was present in equimolar amounts with the amino group. So, it is difficult to anticipate which one of the routes, glycoxidation or lipoxidation, predominates *in vivo*, being CME and CMS, like CML [82], mixed AGEs and ALEs. The possible contribution of myeloperoxidase-catalyzed formation of CM groups from serine interaction with hypochlorite should not be dismissed [83]. In this line, it the possible contribution of PS moieties to glycoaldehyde generation, as precursor of CM groups, might be hypothesized. An alternative source of these compounds could be the oxidative cleavage of the Amadori compounds that would result from glycation of PE or PS. However, glycated PS has not been detected *in vivo*. CME and CMS were detected in RBC and liver mitochondria membranes, and also in urine [54,74,75,81].

The Maillard reaction can generate free radicals through autoxidative processes that, in turn, lead to protein damage [28]. Similarly, these processes, initially described for proteins, may be expanded to aminophospholipids, representing another mechanism for the initiation of lipid oxidation (see Table 2). Accordingly, much evidence indicates that the Maillard reaction on aminophospholipids enhances lipid peroxidation: (i) Autoxidation is usually accelerated by primary amines [52]; (ii) in liposomes, inclusion of PE was found to accelerate the Fe^2+^-dependent peroxidation [52]; (iii) *in vitro*, highest levels of fluorescence intensity were obtained by incubating dilinoleoyl-PE with glucose in oxidative conditions; dipalmitoyl-PE and dilinoleoyl- or dipalmitoyl-PC were ineffective in fluorescence generation independently of the presence/absence of glucose and oxidative conditions [84]; (iv) the presence of a carboxyl group on PS, or two in the case of carboxymethylated serine, may enhance its metal binding capacity, leading to increased metal-catalyzed lipid peroxidation [58]; (v) even in the absence of exogenously added transition metals or free radical generating systems, the formation of AGE-lipids is accompanied by oxidation of the unsaturated fatty acid side chains, suggesting that AGEs formation is a source of free radicals that leads to lipid oxidation [85]; and finally, (vi) the incorporation of glycated PE into LDL facilitates its peroxidation [79].

Despite the fact that non-enzymatic modification of aminophospholipids by glycation, carboxymethylation and lipid peroxidation has been described, they represent only a limited range of the possible products that can likely be formed by the Maillard reaction.

## 5. Biological Significance of MRPs in Aminophospholipids

**Figure 4:**
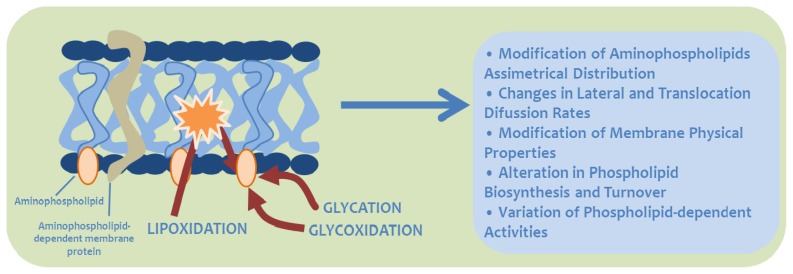
Potential effects of aminophospholipid modification by carbonyl-amine reactions in biological membranes.

The membrane lipid bilayer is not a simple permeability barrier and matrix for protein organization, but a dynamic component capable of initiating and maintaining essential metabolic processes. Considering the presence of free amino groups in aminophospholipids, and their ubiquity in all the biomembranes, *a priori* all of them may be modified by the Maillard reaction. In this way, biological processes involving aminophospholipids could be potentially affected by this non-enzymatic process. Among these, the following may be highlighted [1,3,4,12]: (i) Asymmetrical distribution of aminophospholipids in cellular and different subcellular membranes; (ii) translocation and lateral diffusion in the membrane; (iii) membrane physical properties; (iv) biosynthesis and turnover of membrane phospholipids; and (v) activity of membrane-bound proteins that require aminophospholipids for their function (see Figure 4 and Table 3).

A particularly interesting property of biomembranes is the asymmetric distribution of phospholipids across the bilayer. This is especially evident for the aminophospholipids, PS and PE, which are preferentially located in the membranes’ inner leaflet. Since choline phospholipids are generally more saturated than aminophospholipids, this asymmetry is accompanied by a non-random distribution of fatty acyl side-chains as well. Aminophospholipid asymmetry is controlled by specific mechanisms involving an ATP and Ca^2+^ dependent inward-directed pump known as aminophospholipid translocase, specific for the polar head group [12]. This suggests that the biogenesis and maintenance of the phospholipid asymmetry are important components of membrane homeostasis. So, defects in the normal asymmetric distribution of aminophospholipids may result in altered membrane surface properties that may have important physiological and pathophysiological consequences. As prominent examples of this, one could find the consequences in hemostasis and thrombosis due to exposed PS in activated platelets, and the apparent recognition and sequestration of PS-expressing cells by the reticuloendothelial system [11,39]. Abnormal distributions of aminophospholipids have been also reported for sickle [37] and iron deficient [36] cells, old RBC [35,40], RBC obtained from neonates, patients with chronic myeloid leukemia and diabetes mellitus [41,42], and also in tumorigenic and apoptotic cells [101]. The origin of perturbed aminophospholipid asymmetry in these pathologic cells is unclear, but it might be related to the aminophospholipid nonenzymatic modification described in some of these situations.

PE constitutes a class of lipids, that when dispersed in pure form under physiological conditions, assemble into nonbilayer structures [102]. This contrast to lamellar phase-forming lipids, which readily form hydrated lamellar phases or bilayer structures when dispersed in aqueous media [102]. Lipids with a high propensity to form nonlamellar phases have been implicated in supporting transient local nonbilayer structures, which occur in some cellular processes such as transmembrane movement of ions and large molecules, membrane fusion, membrane enzymatic functions and cell division. However, at present no data are available on the effects of MRP-lipids in these properties.

Theoretically, aminophospholipid biosynthesis and degradation could also be affected by the Maillard reaction: PS is considered both an end product and a biosynthetic intermediate, since it serves as a precursor of PE, given that in eukaryotes, this phospholipid can be formed either by decarboxylation of PS—the major pathway for PE biosynthesis—or through incorporation of ethanolamine into PE via the CDP-ethanolamine pathway. PS synthase is located in the endoplasmic reticulum, whereas PS decarboxylase is strictly confined to the inner mitochondrial membrane, an important finding when accounting aminophospholipid modification in mitochondrial membranes. Finally, PE can be converted into PC by a PE *N*-methyltransferase, constituting a relevant pathway for PC biosynthesis. The consequences of the interference of biosynthetic pathways, if any, that result from the non-enzymatic modification of the polar head of aminophospholipids, remain unknown. How the Maillard reaction—on aminophospholipids—may affect the recognition and transport to other cellular or subcellular membranes by aminophospholipids-binding proteins is also unanswered. In contrast, some data are available from *in vitro* experiments, indicating alterations of the degradative pathway [56]. So, it was described that the hydrolysis of glycated PE by phospholipase A_2_ was not affected, but the hydrolysis by phospholipase C and D was reduced by 50%, in agreement with the fact that glycation affects the polar head but not the acylester bound.

Although several membrane-bound proteins from different biomembranes possibly require aminophospholipids for their function, this fact could be particularly relevant for mitochondrion membranes because several electron transport chain complexes, such as Complex I, Complex III, Complex IV; and the adenine-nucleotide translocator, require binding or interaction with PE [103]. Thus, an increase in the levels of mitochondrial MRPs-lipids, e.g., CME, may lead to serious alterations in mitochondrial respiration and energy production.

The potential effects of these modifications introducing changes in the structural organization of the lipid bilayer and/or changes in the physico-chemical and biological properties of the polar head of aminophospholipids remain to be extensively explored. Nevertheless, considering the serious alterations that can be derived from these non-enzymatic modifications, *a priori*, it can be expected that the levels of these modifications should be found in a low range, at least, in physiological conditions.

## 6. Physiological Significance of MRPs in Lipids

Studies reporting the levels of MRP-lipids in different biomembranes are limited, with RBC membranes being the more extensively studied model. For human RBC and plasma lipids, 1.2% and 2.3%, respectively, of total PE in glycated form, were reported [77]. In another study, concentrations of glycated PE of 9% and 15% were described for RBC and plasma lipids [56]. However, this modification degree, considering that during the preparation and manipulation of lipids prior to analysis Schiff bases were not discharged, may be the result of an overestimation in the percentage of modified PE. Accordingly, the Amadori adducts measurement by GC-MS in aminophospholipids from human RBC revealed a concentration of 0.41 ± 0.11 mmol/mol ethanolamine, representing 0.03% in the glycated form of total PE [58]. With reference to AGE-lipids, only aminophospholipid carboxymethylation, namely CME and CMS formation, was quantified. So, the measurement of CME in human RBCs revealed a value of 0.14 mmol/mol ethanolalmine [54]. CMS was present at an about three times higher concentration (0.44 mmol CMS/mol serine) [58]. So, CME was quantitatively a minor fraction of total ethanolamine, representing only about 0.01% of the modification of RBC membrane phospholipids, in agreement with data reported for phospholipid-MDA adducts representing 0.06%–0.08% of total PE and PS (equivalent to 0.2% of total phospholipids) [40–42].

In urine, CME, CMS, MDA-ethanolamine (MDA-Etn), and MDA-ser were detected [50,51,54,58]; however, only the levels of CME [54] and MDA-Etn [51] were available. So, it was calculated that approximately 2.8 μmol of CME (300 μg) and 0.07 μmol MDA-Etn (5 μg) were excreted daily by a healthy human adult (considering a mean daily creatinine excretion of 1.4 g for a 70 kg subject).

Based on the concentration of CME and MDA-Etn in RBC ghost membranes, ~0.14 and 0.98 mmol/mol of PE, respectively, and the concentration of PE in RBC membranes, ~1 mg/mL of packed RBC (average molecular mass for PE, 800 Da), 0.18 nmol of CME/mL and 1.22 nmol of MDA-Etn/mL of packed erythrocytes can be estimated. Since the average 70 kg subject has about 2 L of packed red cells and ~1% of red cell turnover/day (average red cell life span, 120 days), only about 4 nmol of the daily urinary excretion of CME, and 24 nmol of MDA-Etn, can be attributed to RBC membrane degradation. Anticipating that other modifications may be present in a similar extent, it may be estimated that RBC modifications only represent 1%–5% of the total recovered daily in urine. Consequently more than 95% may arise from the physiological turnover of membrane phospholipids in cells other than red cells, supposing the non-existence of dietary income.

Accordingly, a concentration of 0.11 mmol of Amadori products/mol PE was found in the isolated lipid fractions from rat livers, representing a modification degree of about 0.008%–0.01% [73]. Furthermore, with the mitochondrion being the major site of reactive species production in the cell, it could be hypothesized that aminophospholipids from mitochondrial membranes could be damaged by carbonyl stress. Thus, CME from mitochondrial phospholipids was evidenced in ten mammalian species. The levels observed indicated that CME is quantitatively a minor fraction of total PE, representing only about 0.025%–0.035% modification of mitochondrial membrane phospholipids (considering that all animals are male young adults, and assuming that the mitochondrial phosphatidylethanolamine average is about 35%) [74].

The average inner-membrane surface area per mitochondrion (12.43 μm^2^), when multiplied by the average number of mitochondria per cell (*n* = 1312), shows that the average total inner-membrane surface area in the cell is nearly 11-fold greater than the cell surface area, despite the fact that the mitochondria occupy only 17% of the total cell volume [104]. In the adult rat, the total mitochondrial inner-membrane surface area in the major organs has been found to average 460 m^2^, contributed mainly by skeletal muscle (82% of the total), liver (8%), kidney (5%), heart (3%), brain (1%), and lung (1%) (59). On the other hand, recent studies in liver cell sindicate an average mitochondrial half-life of 3.8 days, corresponding to the destruction of one mitochondrion per cell about every 11 min. Similar conclusions apply in the case of kidney and heart muscle, both indicating that mitochondria are replaced many times during the lifetime of a given cell [104]. Hence, it can be inferred that cellular membranes, and particularly mitochondrial aminophospholipids, might be the major source of MRP-lipids in plasma and urine. In any case, and due to their low levels, it seems likely that MRP-lipids should be considered as biomarkers rather than important effectors of damage in tissues, at least in physiological conditions.

## 7. Significance of MRPs in Aminophospholipids in Aging and Pathological Conditions

Similarly to proteins, MRP-lipids are of interest due to their potential contribution to the physiology of the aging process and age-associated diseases, such as diabetes and atherosclerosis (see Table 4). There is little evidence that demonstrates MRP-lipid accumulation in biological membranes during aging and lifespan. Examining the occurrence of phospholipid-MDA adducts in human RBCs and eye lenses of diverse ages, it was demonstrated that aged RBC showed significantly higher modifications than young erythrocytes (1.5% *versus* 0.2%) [35,40,96], similar to the results obtained for human cataractous lenses [43,44]. Furthermore, lipofuscin—a yellowish brown, lipid-rich, heterogeneous, cytoplasmic granule—shares characteristic autofluorescence with MDA-aminophospholipids, accumulating in a variety of animal tissues, particularly in metabolically active post-mitotic tissues, such as brain and heart, during their aging [34].

In another comparative-physiological approach, it was demonstrated that mitochondrial CME levels correlated with lifespan, *i.e.*, levels of CME in short-lived are lower than those of long-lived mammals [74]. In this line, a previous study [109] which evaluated the effects of the aging rate of different mammalian species on the skin collagen concentration of pentosidine, a biomarker of glycoxidative protein damage, showed that although the highest pentosidine accumulation rates were observed in short-lived species, the absolute levels of pentosidine were smaller than those of long-lived species, similarly to the finding of low CME levels in short-lived species. All these data raise the question about the importance of absolute levels of protein and lipid oxidation markers before their accumulation rates. It is conceivable that short-lived species may exhibit higher rates of CME formation, but given the short half-life of aminophospholipids, rates of turnover will presumably be the dominant factor determining the levels of mitochondrial CME. Thus, the observed trends should be ascribed to interespecies differences in CME accumulation and/or phospholipid turnover rates. However, several aspects such as the urinary excretion as the expression of the turnover of damaged aminophospholipids, the implications of CME in mitochondrial and cellular metabolism, and the relationship of CME in mitochondria with the rate of aging remain to be explored.

Diabetes induced a 2.5-fold increase of glycated aminophospholipids in the liver, and 0.025% *vs.* 0.01%, in diabetes *vs.* control [73]. In a LC-MS-based study, it was reported that 1.2% and 2.3% of total PE were glycated in human RBC and in plasma lipids, respectively. Furthermore, these values were increased to 10% and 16% in diabetic patients, representing a 10-fold difference [77]. In these studies, special care was taken to not discharge Schiff bases during prechromatographic sample preparation. The Amadori products measurement by GC/MS showed about a three-fold increase in concentrations of GE in RBC lipids from diabetic patients relative to healthy controls [58]. In this line, it was reported that the concentration of phospholipid-MDA crosslink in RBC membranes was elevated approximately three-fold in diabetic subjects [41].

AGE-proteins play a key role as causative agents in the pathogenesis of diabetic complications [110]. In agreement with this concept, concentrations of CML and other AGEs are elevated in different tissues of diabetic patients, correlating with the severity of the chronic complications. Since patients that participated in the referred study were free of complications [58,81], the lack of differences in CME levels, as well as CML, between control and patient populations was not an unexpected finding. Furthermore, and consistent with RBC data, neither urine CME nor CML concentrations were significantly increased by diabetes. So, further studies are needed to examine levels of carboxymethylated lipids in diabetic patients with more extensive vascular complications, predicting the existence of differences. In fact, it should be noted that a four-fold increase of MRP-lipids in LDL from diabetic patients *vs.* healthy subjects was reported by using enzyme-linked immunosorbent assay (ELISA), suggesting a greater increase in Maillard-type modification of aminophospholipids by diabetes [72], although responsible structure/s were not characterized.

In this line, LDL glycation has been suggested to be responsible for the increased susceptibility to atherogenesis of diabetes subjects [111,112]. Thus, a recent report has indicated that PE glycation of LDL is accounted for completely by the known effect of LDL glycation on macrophage uptake, cholesteryl ester and triacylglycerol accumulation. Therefore, there is an increase in LDL atherogenic potential in the diabetic status [79]. Furthermore, and contributing to the pathogenesis of diabetic vascular complications, the interaction of RBC from diabetic rats with a receptor termed RAGE (receptor for AGEs) has been described [113]. A recent report indicates the recognition of “carboxymethyl” structures by RAGE [114]. This interaction could contribute to the shortened half-life of diabetic RBC when injected into normal animals, leading also to a localized increase in oxidative stress in the vascular wall. The cause of this interaction could be explained by the exposition to the outer layer of the modified aminophospholipids, as previously described. However, the AGE structure(s) on the RBC surfaces mediating their interaction with the RAGE have not been characterized.

## 8. Summary

Membrane composition (phospholipids classes’ distribution and fatty acid profile) is strictly and dynamically regulated. The highly unsaturated fatty acids present in cellular membranes are the most susceptible macromolecules to oxidative damage in cells, and this sensitivity increases as a function of the number of their double bonds. Carbonyl-amine reaction-derived molecular damage is a natural consequence of aerobic life. Current data indicate the extension of carbonyl-amine reactions to aminophospholipids. Despite the fact that non-enzymatic modification of aminophospholipids by glycation, carboxymethylation and lipid peroxidation has been described, they represent only a limited range of the possible products that can likely be formed by the Maillard reaction. Due to their important role in cellular homeostasis, further studies are needed to elucidate the potential implications of these reactions on basic life processes.

## Figures and Tables

**Table 1:** Summary of studies addressing the characterization of non-enzymatic aminophospholipid modification by carbonyl-amine reactions.

Experimental model	Analytical approach	Structural characterization	Ref.
***In vitro*Studies**			
Peroxidation of arachidonate and docosahexaenoate + synthetic dipalmitoyl phosphatidylethanolamine	Fluorimeter	Fluorescence chromophores (Ex 360 nm, Em 430 nm)	[31]
Rat liver mitochondrial and microsomal fractions peroxidized *in vitro*	Fluorimeter	Fluorescent chromophores (Ex 365–370 nm, Em 435–440 nm)	[47]
Methyl arachidonate/methyl linolenate/methyl linoleate/Malondialdehyde + phosphatidylethanolamine/phosphatidylserine (PE/PS)	Fluorimeter	Fluorescent chromophores (Ex 365 nm, Em 435 nm)	[32]
Malondialdehyde + Red blood Cells (RBC)	Fluorimeter	Fluorescent chromophores (Ex 390–400 nm, Em 460 nm)	[35]
Lipid peroxidation of rat liver microsomes	Fluorimeter	Fluorescent chromophores (Ex 359 nm, Em 430 nm)	[48]
Lipid peroxidation-derived compounds	Fluorimeter	Fluorescent chromophores (Ex 360 nm, Em 435 nm)	[33]
Malondialdehyde + primary amines	Fluorimeter	Identification of 1–4-Dihydropyridine-3,5-Dicarbaldehydes as model of fluorescent components in lipofuscin (Ex 375–405 nm, Em 435–465 nm)	[49]
4-Hydroxynonenal (4-HNE) + microsomes/mictochondria/phospholipids (PS and PE)	Fluorimeter	Fluorescent chromophores (Ex 360 nm, Em 430 nm)	[45]
Malondialdehyde (MDA) + serine	Nuclear magnetic resonance (NMR) and (high performance liquid chromatography (HPLC)	*N*-2-(Propenal)serine	[50]
MDA + ethanolamine	NMR and HPLC	*N*-(2-propenal)ethanolamine	[51]
Mixed liposomes of l-alpha-dilinoleoyl-phosphatidylcholine (DiLinPC) and l-alpha-Dilinoleoylphosphatidylethanolamine (DiLinPE) in oxidative conditions	Fluorimeter and oxygen consumption monitored polarographically with a Clark-type oxygen probe	Fluorescent chromophores (Exc 360 nm, Em 430 nm)	[52]
Glucose + PE and PS	Liquid chromatography-electrospray ionization-mass spectrometry (LC-ESI-MS and TLC)	Glycated aminoglycerophospholipids	[53]
Lens + oxidative conditions	Fluorimeter and TLC	MDA:aminophospholipid adducts (Ex 360 nm, Em 470 nm)	[44]
Glycoxidation and autoxidation of PE from RBCs	Gas chromatography-mass spectrometry (GC-MS)	Carboxymethyl-ethanolamine (as marker of carboxymethyl-phosphatidylethanolamine, CM-PE)	[54]
Glucose + phosphatidylethanolamine	Gas-liquid chromatography-mass spectrometry (GLC-MS) and HPLC with diode-array detection (DAD)	1-deoxy-1-(2-hydroxyethylamino)-*D-*fructose derivatives	[55]
Glucose + PE/phosphatidylcholine (PC)-PE liposomes	Thin layer chromatography (TLC), HPLC, NMR, fast atomic bombardment (FAB)-MS	Deoxy-*D*-fructosyl PE	[56]
(2-aminoethyl)phenetydylphosphate and (2-aminoethyl)ethylphosphate as model of aminophospholipids + carbohydrates	HPLC, NMR	Aminophospholipid glycation	[57]
PE and PS + glucose	GC-MS	*N*-(glucitol)ethanolamine and *N*-(carboxymethyl)serine	[58]
Human low density lipoprotein (hLDL) + p-hydroxyphenylacetaldehyde (p-hydroxyphenylacetaldehyde (pHA), product of l-tyrosine oxidation by the myeloperoxidase system of macrophages)	GC-MS	pHA-ethanolamine	[59]
Glucose and 3-Deoxyglucosone + PE	GLC-MS and HPLC-DAD	Formation of a phospholipid-linked pyrrolecarbaldehyde	[60]
Carbohydrates + PE	LC-ESI-MS, HPLC-DAD, and NMR	PE-derived Amadori compounds	[61]
PE + 4,5(E)-epoxy-2(E)-heptenal (secondary product of lipid peroxidation)	GC-MS, HPLC-MS, NMR	Phosphatidylethanolpyrroles and phosphatidylethanol-2-(1-hydroxypropyl)pyrroles	[62]
PE + 13-hydroperoxyoctadecadienoic acid and other oxidized poly unsaturated fatty acids (PUFAs) followed by phospholipase *D*-mediated hydrolysis	LC-MS and NMR	*N*-(hexanoyl)ethanolamine	[63]
Different species of PE + 4-hydroxy-*trans*-2-nonenal (4-HNE)/4-hydroxydodecadienal (4-HDDE)/4-hydroxyhexenal (4-HHE)	GC-MS, TLC, HPLC, and NMR	Aldehydes-PE. Different PE species are differently targeted by fatty aldehydes	[64]
PE + Isoketals (IsoK)	LC-ESI-MS	IsoK-PE pyrrole adducts and IsoK-PE Schiff base adducts	[65]
Fatty aldehydes released from plasmalogens after oxidation of cerebral cortex homogenates	GC-MS	*N*-heptadecyl-PE	[66]
PE + Glucose + Potential “antiglycative” compounds (protein glycation inhibitors, antioxidants, vitamins, *etc.*)	LC-ELSD-MS	Amadori-PE. Pyridoxal 5′-phosphate and pyridoxal/vitamin B6 derivatives) are the most effective antiglycative compounds	[67,68]
Acetoacetate + brain aminophospholipids	TLC and spectrophotometry	UV spectroscopy at 280 nm	[69]
Acetaldehyde-PE	Density functional theory study	Schiff base formation between PE and acetaldehyde	[70]
Liposomes and human high density lipoprotein (hHDL) particles in oxidative conditions	LC-MS	Isolevuglandins (IsoLGs)-, MDA-, 4.HNE-, *N*-Acyl-, and *N*-carboxyacyl-PEs	[71]
***In vivo*Studies**			
hLDL	Fluorimeter	Fluorescent chromophores AGEs-lipids (Ex 360 nm, Em 440 nm)	[72]
Rat liver aminophospholipids	GC-MS	Amadori aminophospholipids (as 5-(hydroxymethyl)-2-furfuraldehyde; 5-HMF)	[73]
Liver mitochondria from mammalian species	GC-MS	Carboxymethyl-ethanolamine (as marker of CM-PE)	[74]
hRBCs	TLC, HPLC, NMR, FAB-MS	Deoxy-*D*-fructosyl PE	[56]
RBC membranes	GC-MS	Glycation and carboxymethylation of aminophospholipids (PE and PS)	[75]
Glucose/lactose + PE; foods and biological samples Presence in foods (e.g., infant formula, chocolate) and in rat plasma	HPLC-UV (labeling with 3-methyl-2-benzothiazolinone hydrazone)	Glycated-PE and lactose-PE	[76]
RBC and LDL	LC-MS and NMR	*N*-(hexanoyl)ethanolamine	[63]

**Table 2:** Summary of studies on physico-chemical effects of non-enzymatic aminophospholipid modification by carbonyl-amine reactions.

*In vitro* or *in vivo* experimental model	Analytical approach	Marker	Finding	Ref.
Rat and human urine	NMR and HPLC	*N*-2-(Propenal)serine	Direct evidence for oxidative decomposition of phospholipids by lipid peroxidation	[50]
Rat and human urine	NMR and HPLC	*N*-(2-propenal)ethanolamine	Direct evidence for oxidative decomposition of phospholipids by lipid peroxidation	[51]
Glucose + PE/hLDL	Fluorimeter	Fluorescent lipid advanced glycosylation (Ex 360 nm, Em 440 nm)	Increase of fluorescence associated with the progressive oxidative modification of unsaturated fatty acid residues	[72]
Lipids (PE and PS) and hLDL-advanced glycosylation	GC-MS	4-hydroxyhexenal and 4-hydroxynonenal	Lipids-AGE formation in close proximity to unsaturated fatty acyl groups leads to lipid peroxidation	[85]
Unilamellar vesicles with PE and PC + glyceraldehyde	Time-resolved fluorescence spectroscopy		Aminophospholipid glycation increases the head-group hydration and lipid order in both regions of the membrane and lipid glycation is accompanied of lipid oxidation	[86]
Atherosclerotic plaques collected from both diabetic and non-diabetic subjects	LC-ESI-MS	Glycated PE	Glycated aminophospholipids are the major LDL glycation products and increase LDL susceptibility to oxidation	[87]
Model systems and egg yolk products	LC-ESI-MS	Identification of PE-linked glucosylamines (Schiff-PE), Amadori products (Amadori-PE), 5-hydroxymethylpyrrole-2-carbaldehydes (Pyrrole-PE), and carboxymethyl- (CM-PE) as well as carboxyethyl-(CE-PE) derivatives	Possible influence on emulsifying properties and oxidation resistance	[88]
Amadori-PE + linoleic acid	LC-MS and colorimetry	TBARs and lipid hydroperoxides	Glycated-PE trigger lipid peroxidation via free radical reactions	[89]
RBCs from diabetic and healthy individuals	LC-ESI-MS	Schiff-PEs and Amadori-PEs, and detection of pyrrole-PE, CM-PE and CE-PE	Increase in diabetes; glycated PE promotes lipid peroxidation of biomembranes	[90]
Different species of PE + 4-HNE/4-HDDE/4-HHE	GC-MS, TLC, HPLC, and NMR	Aldehydes-PE	Different PE species are differently targeted by fatty aldehydes: the higher their hydrophobicity, the higher the amount of adducts made	[64]
PE/PC monolayers + 4-HNE	Alternating current (AC) polarography	Physico-chemical state of a condensed PE-containing phospholipid monolayer and its interaction with apo A-I	4.HNE-PE does not alter monolayer stability, but decreases apo A-I insertion into the monolayer	[91]
PC/PE mixture + Glucose + isolated membrane proteins	Lipid-protein interactions	Amadori-PE and Amadori-proteins, and lipid-protein interaction parameters	Lipid glycation decreases the affinity of lipids for membrane proteins, induces structural rearrangements in the protein that makes it more sensitive to thermal unfolding and decreases the affinity between proteins and the surrounding phospholipids.	[92]
PE + Glucose + oxidative conditions	LC-ESI-MS	Glycated-PE + oxidation products	Oxidation of glycated-PE occurred more quickly than the oxidation of non-glycated-PE probably because of the existence of more oxidation sites derived from glycation of polar head group.	[93]
PE + Glucose + oxidative conditions	LC-MS-MS	Identification of free radicals in oxidized and glycoxidized PE	Presence of several sites susceptible to oxidation in glycated-PE which may be responsible for the increase in the oxidative reaction rate occurring in glycated compounds	[94]

**Table 3:** Summary of studies on biological effects of non-enzymatic aminophospholipid modification by carbonyl-amine reactions.

Experimental model	Analytical approach	Marker	Finding	Reference
MDA+RBCs and *in vitro* lipid peroxidation of RBC	TLC	MDA:phospholipid adducts	Lipid peroxidation and MDA accumulation disturb organization of PS and PE in the human erythrocyte membrane bilayer	[38]
Erythrocytes of phenylhydrazine-treated rats	TLC	MDA:phospholipid adducts	Externalization of PS and PE in the membrane bilayer and hypercoagulability	[39]
Glucose-treated RBC	TLC	MDA:phospholipid adducts	Increase adduct formation and osmotic fragility in human RBCs	[42]
hRBCs from different age groups + MDA or H2O2 treatment		Aminophospholipid translocase activity	Decrease with age (defects in endogenous lipid asymmetry observed in aged human RBCs may be due to altered activity of the translocase)	[95]
Lipid extracts from platelet incubated PE + PS + 4-HNE	LC-MS	PE-4-HNE compounds	Formation in cell membranes that could alter the phospholipase-dependent cell signalling	[46]
Glycated-PE LDL + THP1 cells (macrophages)	Cell culture, LC-ESI-MS	Glycated PE	Glycated-PtdEtn promotes macrophage uptake of LDL and accumulation of cholesteryl esters and triacylglycerols	[79]
Oxidized-LDL + platelets	TLC	Aldehyde-PE	Modified PE as the active component of oxidized LDL particles elicit a pronounced prothrombotic response by increasing the activity of the platelet prothrombinase complex	[96]
PE + 4-HNE and 4-HDDE (4-hydroxy-2E,6Z-dodecadienal)	TLC and GLC	4-HNE-PE and 4-HDDE-PE	Modified PE is poor substrate for secreted phospholipase A2	[97]
Human Plasma	TLC and GLC	4-HNE-PE and 4-HDDE-PE	Potential alteration of phospholipid-dependent cell signaling	[97]
Amadori-PE + endothelial cells (HUVEC)	Cell culture	Cell proliferation, migration, and tube formation, and secretion of matrix metalloproteinase 2 (MMP-2)	Glycated-PE promotes vascular disease as a result of its angiogenic activity on endothelial cells	[98,99]
Human blood platelets	GC-MS	4-HHE-, 4-HNE-, and 4-HDDE-PE	*In vivo* identification. Increase of platelet aggregation	[100]

**Table 4:** Summary of studies on physiological and pathological effects of non-enzymatic aminophospholipid modification by carbonyl-amine reactions.

Experimental model	Analytical approach	Marker	Finding	Reference
Aged RBC	Fluorimeter	Fluorescent chromophores (Ex 390–400 nm, Em 460 nm)	Increase with aging Altered physical and biochemical properties of aging cells (polymerization of plasma membrane proteins)	[35]
Lipids extracts from different tissues (heart, brain, liver, testis, kidney, adrenal cortex)	Fluorimeter	Fluorescent chromophores	Increase with aging, and in pathological conditions (e.g., diabetes, hyperlipidemia)	[15,34]
RBC fromiron-deficient infants and animals	Thin Layer Chromatography (TLC)	Schiff’s base adduct due to reaction MDA + PS/PE	Decrease RBC survival	[36]
RBC from the “sickle cell disease”	Fluorimeter and TLC	Fluoresecent chromophores & MDA:aminophospholipid adducts	Increase adduct formation in sickle cell disease	[37]
Lipid extracts of the human cataractous and normal lenses	Fluorimeter and TLC	Fluoresecnt chromophores (Ex 365 nm, Em 460 nm) and MDA: aminophospholipid adducts	Increase in human senile cataract	[43]
Aged human RBC membranes	Fluorimeter and TLC	Fluorescent chromophores & MDA: aminophospholipid adducts	Increase with aging	[40]
RBC from diabetic patients	TLC	MDA:phospholipid adducts	Increase adducts in diabetes	[41]
humanRBC (hRBC) from different age groups + MDA or H_2_O_2_ treatment		Aminophospholipid translocase activity	Decrease with age (defects in endogenous lipid asymmetry observed in aged human RBC may be due to altered activity of the translocase)	[96]
LDL from diabetic patients	Fluorimeter		Increase in diabetes	[72]
Rat liver aminophospholipids in streptozotocin-induce diabetic rats	GC-MS	Amadori aminophospholipids (as 5-(hydroxymethyl)-2- furfuraldehyde; 5-HMF)	Increase in diabetes	[73]
RBC and plasma from diabetic and control subjects	LC-ESI-MS	Glycated aminophospholipids	Increase in diabetes	[77]
Urine from diabetic and control subjects	GC-MS	Carboxymethylethanolamine (as marker of CM-PE)	No increase in diabetes	[54]
Liver mitochondria from mammalian species	GC-MS	Carboxymethyl-ethanolamine (as marker of CM-PE)	CM-PE formation at mitochondrial level is associated with animal lifespan	[74]
hRBC from diabetic and control subjects	GC-MS	*N*-(glucitol)ethanolamine & *N*-(carboxymethyl)serine	Adducts formed *in vivo* and increased in diabetes	[58]
hLDL from plasma and atherosclerotic aorta	GC-MS	pHA-ethanolamine	Increase of pHA-PE in human atherosclerotic intima	[59]
RBC from diabetic and healthy individuals	LC-ESI-MS	Schiff-PEs, Amadori-PEs, pyrrole-PE, CM-PE and CE-PE	Increase in diabetes; glycated PE promotes lipid peroxidation of biomembranes	[90]
Amadori-PE of a lipid extract from diabetic plasma	QTRAP LC-MS-MS	Amadori-PE	Increase in diabetes	[105]
Plasma from: healthy volunteers, chronic hemodyalisis patients, Type II diabetic patients without chronic hemodialysis, and Type II diabetic patients with chronic hemodialysis	HPLC-MS-MS	Amadori-PE	Increase of Amadori-PE in diabetes with or without chronic hemodialysis	[106]
Plasma from streptozotocin-diabetic rats	LC-ELSD-MS	Amadori-PE	Increase in diabetes and decrease in streptozotocin-induced diabetes and pyridoxal-treated rats	[67,68]
Retinas of streptozotocin-induced diabetic rats	GC-MS	4-HHE-, 4-HNE-, and 4-HDDE-PE	Increase in diabetic animals	[101]
hRBC and blood plasma from healthy subjects and diabetic patients	LC-MS-MS	CM-PE and CE-PE as AGE-PE, and Amadori-PE	No significant differences were observed in AGE-PE in RBC and plasma, whereas Amadori-PE concentrations were higher in diabetic patients	[107]
Blood plasma, kidney, RBCs, liver, pancreas, cerebrum, and cerebellum from streptozotocin-induced diabetes rats	LC-MS-MS	Amadori-PE	Increase in diabetes. Amadori-PE(18:0–20:4) is the PE species that acts as the most sensitive indicator	[108]
